# Evaluation of the operational efficiency and policy innovation of chinese happy farmhouse: From the perspective of business entities

**DOI:** 10.1371/journal.pone.0339582

**Published:** 2026-01-02

**Authors:** Tian Tian, Hong Yan, Youli Xu

**Affiliations:** 1 School of Economics and Management, Zhejiang Shuren University, Hangzhou, China; 2 School of Public Affairs, Zhejiang Shuren University, Hangzhou, China; Islamic Azad University Urmia Branch, IRAN, ISLAMIC REPUBLIC OF

## Abstract

The development of Happy Farmhouse tourism in China faces significant challenges of homogenized competition and low operational efficiency. This study aims to evaluate the operational efficiency of these farmhouses and compare the performance across three distinct business entity models: individual farmer, farmer participation, and company cluster. To this end, the study employs a three-stage Data Envelopment Analysis (DEA) model on panel data (2014–2023) from 50 farmhouses in Huzhou, China, to measure technical, scale, and managerial efficiency. Results reveal steady improvements in overall performance but significant efficiency disparities, with company clusters demonstrating the highest efficiency, followed by farmer participation models, and individual farmers demonstrating the lowest. The primary reasons for this disparity are identified as differences in scale efficiency and talent supply. Consequently, our findings provide actionable insights for optimizing rural tourism’s role in sustainable development and rural revitalization, with policy recommendations emphasizing talent recruitment, cluster-based management, and targeted support for different business models.

## 1. Introduction

The accelerating global urbanization process has precipitated rural decline as a worldwide challenge, prompting nations to reconstruct rural development trajectories through policy innovation [1]. International exemplars such as the UK’s “Rural Central Village Construction” and France’s “Rural Revitalization Plan” demonstrate that revitalizing endogenous rural dynamics must be fundamentally rooted in local resource endowments [2]. Within China’s context, this challenge manifests acutely through the urban-rural dual-structure contradiction. Despite remarkable industrialization and urbanization achievements, rural areas persistently confront systemic challenges including industrial hollowing-out, infrastructural deficits, and ecological fragility [3–4]. Statistical evidence revealing an urban-rural income disparity ratio of 2.56:1 in 2020 epitomizes the severity of this development imbalance [5].

In this context, rural tourism, especially the “Happy Farmhouse” model originating from the 1987 Chengdu Peach Blossom Festival, has emerged as a strategic measure to bridge the urban-rural gap [6]. By 2022, official statistics documented over 2.2 million registered farmhouse enterprises generating annual revenues surpassing ¥800 billion, demonstrating significant quantitative success. Nevertheless, this expansion masks multidimensional challenges that threaten the sector’s sustainability: homogenized competition precipitates suboptimal operational efficiency [7], environmental carrying-capacity overextension induces ecological degradation [8], and inadequate service standardization constrains quality assurance [9].

While the Happy Farmhouse sector has proliferated, a critical research gap remains at the micro-level of operational performance from the perspective of business entities. Existing studies have largely overlooked how different governance structures—specifically the individual farmer, farmer participation, and company cluster models—fundamentally shape managerial practices and efficiency outcomes. The core problem, therefore, is not merely the existence of inefficiency, but a lack of understanding of its extent, its drivers across different business models, and how policy can be innovated to address these specific inefficiencies.

To this end, this study aims to explore the level of operational and managerial efficiency of Happy Farmhouses and how it has evolved. Further, it investigates what are the primary sources of efficiency disparities among the three dominant business entity models. Moreover, the at what level the specific policy innovations and managerial strategies enhance the sector’s overall efficiency and contribution to rural revitalization. For these objectives, our research employs a three-stage Data Envelopment Analysis (DEA) model on panel data (2014–2023) from 50 Happy Farmhouses in Huzhou, China, to measure technical, scale, and managerial efficiency while controlling for environmental factors. The significance of this study is twofold. Theoretically, it expands the analytical framework for rural tourism research by introducing a rigorous, micro-level efficiency analysis from a business-entity perspective. Practically, the findings provide actionable insights for optimizing operational practices and inform targeted policy innovations, thereby supporting the high-quality development of rural tourism and its role in China’s broader rural revitalization objectives. The rest of the study is organized as follows: Literature review and research design are discussed in sections 2 and 3. Section 4 explains the results and discussion. Study conclusions and suggestions are illustrated in section 5.

## 2. Literature review

### 2.1. Research progress of Happy Farmhouse

The mainstream of research results covers management type, development mode and management performance of the happy farmhouse. Academic interest in Happy Farmhouses has evolved in tandem with policy shifts, with initial research peaks occurring between 2008 and 2011, followed by a renewed surge from 2021 onward, aligned with the deepening of China’s national rural revitalization strategy.

#### 2.1.1. Study on the types of farmhouses.

Fan [10] divided happy farmhouses into nine types according to different types of operations, including sightseeing farm, urban park, agricultural park, leisure farm and farmhouse. Ding & Sun [11] divided Leisure Agriculture into six types according to different management structures: leisure planting, leisure forestry, leisure animal husbandry, leisure fishery, leisure sideline and leisure ecological agriculture. The various functions can be divided into seven types: sightseeing farm, leisure farm, science and technology farm and environmental farm. According to Chen, three different classification standards can be seen [12]. The first is according to the types of independent management, farmers as the leading operators, company-led participation of farmers, companies and communities, farmers’ cooperation, leasing, village committee-led enterprises, individual operations, equity farming enterprises and multi-stakeholder cooperation. The second classification standard is defined according to the actual situation and the specific local human customs or natural resources. The third criterion is to classify the benefits brought by Happy Farmhouse in various aspects, such as economic and cultural.

From the point of view of management subject, taking Zhejiang province as an example, Shen and Yu [13], believe that the factors affecting the management mode of happy farmhouse in Zhejiang mainly include the government and the operators, which are reflected in the weak infrastructure of happy farmhouse and the low level of management, such as the strong modern flavor of farmhouse, and give the relevant suggestions. Li [14], points out that there are many factors affecting the operation mode of Neijiang Happy Farmhouse. Still, the critical one is the government support, followed by investors, Happy Farmhouse owners and service personnel. It is believed that as long as corresponding measures are taken under the active guidance of the government, Neijiang’s rural happy farmhouse will be developed better. Yao [15] based on the development status of happy farmhouse tourism in Hefei and the excellent happy farmhouse tourism models, proposes that the factors affecting the development of the local happy farmhouse tourism model are the government’s support, enterprise training and network publicity, and the development is carried out according to local conditions through the government’s strong support, increased publicity and good operation norms.

#### 2.1.2. Development of Happy Farmhouse.

Guo [16], in a comparative study of leisure agriculture in Taiwan and the Chinese mainland, concludes that the mainland’s leisure agriculture has such limiting factors as similar products without characteristics, lagging research planning, incomplete infrastructure, extensive operation and management, single function, insufficient initial funds, and insufficient publicity. Bai [17] takes Guiyang city as an example and points out that Guizhou’s leisure agriculture is weak in brand promotion and industrial cluster scale expansion. Wang and Zhang [18] point out that Guizhou’s leisure agriculture still has problems such as serious product homogeneity, lack of cultural characteristics, weak brand awareness and extensive resource development methods. Xu [19] investigates the happy farmhouse in Guizhou, and finds that there are four problems in service quality and level to be improved, infrastructure construction to be strengthened, supervision and management mechanisms to be imperfect, and a lack of outstanding characteristics of the happy farmhouse brand.

Scholars have revealed the problems encountered in the development of rural tourism and also proposed optimization paths from multiple dimensions. For instance, Han et al. [20] describe specific characteristics of upgrading the consumption structure, and took Bengbu Farmhouse as an example to suggest cultivating customer groups, building brands, and characteristic positioning. Jin [21] points out that to meet Chinese residents’ tourism demand after consumption upgrades, it is necessary to start with infrastructure, a tourism complex, and a quality tourism service. Yuan [22] proposes an innovative path of happy farmhouse development mode: improve infrastructure construction, optimize industrial structure and activate the operation mechanism of happy farmhouse, to promote the sustainable development of rural tourism. Wang [23] points out that there are still problems, such as complex complaints, blindly following the trend, a lack of cultural connotation and low benefits in the operation of farm happy farmhouse, and puts forward corresponding countermeasures. Dong [24] analyses the current situation and problems of Chengdu’s rural tourism development and gives a development strategy.

#### 2.1.3. Research on the management efficiency of Happy Farmhouse.

When the global tourism economy reaches a particular scale, the pursuit of tourism development quality and efficiency becomes a deeper problem that needs to be solved. Many researchers studied tourism efficiency and influencing factors at different scales from different angles. Marrocu and Paci [25] selected human capital, social capital, technological capital, and tourism flow as basic variables to study the total productivity of tourism factors and its spatial spillover effect in 199 regions of the European Union. Hadad et al. [26] evaluated the economic efficiency of tourism in 34 developed countries and 71 developing countries, and it shows that globalisation and accessibility are crucial to the efficiency of tourism in developing countries. At the same time, labor productivity has an essential impact on the overall efficiency of tourism globally. Predonu [27] took Romania as a case study to discuss the significance of tourism development and internal mechanisms to improve national economic and social efficiency. Soysal Kurt [28] used the CCR-DEA method to estimate the relative tourism efficiency of 29 European countries in 2013, taking tourism cost, number of employees and number of reception beds as input variables and tourism income, number of tourists and number of stay days as output variables. The results show that the number of countries where tourism is relatively effective and ineffective is 16 and 13, respectively. Gursoy and Goral [29] measured and compared the tourism efficiency index of 129 countries in the world based on the DEA super efficiency model. Corne and Peypoch [30] first used DEA to evaluate the tourism performance of 13 tourist destinations in France. Then, based on the tourism attraction theory, fuzzy set qualitative comparative analysis (fsQCA) was used to discuss the number of monuments, the number of museums, whether there are beaches and ski resorts, and their impact on the efficiency of tourist destinations. Radovanov et al. [31] investigated the tourism development efficiency of 27 EU member states and 5 Balkan countries from the perspective of sustainable development by using panel data from 2011 to 2017. The study shows that the average tourism efficiency of the 15 EU countries is the highest, the Balkan countries are higher, and the tourism efficiency of the later member states is the lowest. At the same time, it is found that the sustainability of tourism development, the share of GDP, the number of tourists and the income of inbound tourism have significant effects on tourism efficiency. Jiang et al. took the rural tourism in the outer suburbs of Beijing as the research object and used the DEA-Malmquist index model to conduct a comprehensive evaluation of the rural tourism efficiency, and found that the expenditure on agriculture, forestry and water affairs, employees, per capita GDP and the number of tourism resources had significant positive influences on the tourism efficiency. It also gives development suggestions from accelerating the integration of rural tourism resources, continuously increasing the financial investment to support agriculture, and strengthening the education and training of practitioners. Dong [32] measured and analysed the efficiency of the six different scenic areas in China and concluded that the efficiency of East China and Southwest China was better, while that of Northwest and Northeast China was worse. In addition, Liu et al. [33], Xu [34], Luo [35] and Yang [36] took Anhui Province, Jiangxi Province, Li minority areas in Hainan Province and Jilin Province as research objects, respectively, to explore the efficiency of rural tourism poverty alleviation in the above regions and put forward suggestions for optimizing development.

#### 2.1.4. Review of research.

The existing research has made rich achievements in the types of farmhouse operation, development problems and operation efficiency. Compared with previous studies, this paper makes two improvements and explorations. First, regarding research scope, while previous efficiency assessments predominantly focused on macro- and meso-level analyses, this study shifts to a micro-level perspective by specifically targeting happy farmhouse operations. This approach fills a critical gap in the literature, which has largely overlooked efficiency evaluations at the firm level within China’s micro level.

Second, in methodological rigor, conventional Data Envelopment Analysis (DEA) models often neglect external environmental factors that significantly influence operational performance, leading to biased estimates. To address this limitation, we adopt the three-stage DEA framework proposed by Fried et al., which systematically disentangles the effects of environmental variables and statistical noise. Our analysis leverages panel data (2014–2023) from 50 happy farmhouses in Huzhou, Zhejiang Province, sourced from the Zhejiang Provincial Department of Culture and Tourism. This dataset enables a granular examination of comprehensive efficiency, pure technical efficiency, and scale efficiency across diverse operational models. Further analytical techniques—including slack value analysis and super-efficiency modelling—provide actionable insights for sectoral governance. Key contributions of this study include: firstly, Enhanced Measurement Accuracy. Integrating Stochastic Frontier Analysis (SFA) into the DEA framework isolates environmental confounders (e.g., policy support, regional GDP) and stochastic disturbances, yielding purified efficiency metrics that reflect actual managerial performance. Secondly, introducing the super-efficiency model enables horizontal comparison between business entities whose technical efficiency reaches the optimal level, to identify benchmark cases. Thirdly, dividing the happy farmhouses into three types: shareholder cooperative type, enterprise cluster type and individual family type, reveals how the governance structure regulates the relationship between economies of scale and technical efficiency.

### 2.2. Efficiency evaluation method

The analytical methods to measure efficiency can be divided into two categories: the financial index analysis method and the boundary analysis method.

Financial index analysis is a more traditional evaluation method, divided into a single financial index evaluation method and a multi-financial index evaluation method. Its advantage is that it is simple to use and easy to understand. Still, selecting financial indicators is relatively arbitrary, which cannot overcome the collinearity and correlation interference among financial indicators, and the evaluation result may be wrong. Therefore, with the continuous development of enterprise efficiency theory research and the abundance of measurement tools, efficiency evaluation research has gradually changed from traditional financial index analysis to boundary analysis.

Boundary analysis method, or frontier analysis method, can be divided into parametric method and non-parametric method according to different methods of measuring technical efficiency. The Stochastic Frontier Approach (SFA) typically represents the parametric method. It uses the production function or cost function as the evaluation basis. Random interference factors in reality are included to deal with inefficiency problems in production behavior. Data Envelopment Analysis (DEA) is the most commonly used non-parametric frontier efficiency analysis method. Its evaluation mode adopts linear programming technology to replace the production function in economics, and takes the production frontier as the basis for measuring the point of production efficiency. The total efficiency is divided into the technical efficiency, which discusses the relationship between input and output, and the allocative efficiency, which discusses the combination of input factors. Technical efficiency refers to the ability to produce the maximum yield under the fixed amount of input factors, and can be further divided into pure technical efficiency and scale efficiency. Allocative efficiency refers to the ability to adjust the input-output ratio optimally under the relative price of production technology input factors. Given the different research purposes, some scholars have expanded the DEA model.

Li & Fan [37] compared DEA and SFA, and summarized the similarities and differences between them, including that both belong to frontier measurement methods, their everyday basis is the distance function, and they measure technical efficiency based on constructing the production frontier. The differences are as follows: the complexity of basic assumptions and model expansion of the two models are different, the interpretation and processing methods of actual output are different, the methods of constructing production frontier are different, the stability of calculation results are different, the relevant economic information obtained is different, and the methods of analyzing the factors affecting efficiency are different. They also suggest combining the two approaches or the measurements of both approaches when it is impossible to determine which is better for the problem. That is, random nonparametric method or a combined comprehensive evaluation method.

The three-stage DEA model, proposed by Fried et al., combines DEA and SFA to measure efficiency comprehensively. The first stage is the initial efficiency value calculation. In this stage, the traditional DEA model is used to calculate each decision-making unit’s efficiency value, and each input variable’s relaxation value is obtained. The efficiency value may be inaccurate due to environmental variables, statistical interference and management efficiency. The second stage is the calculation of the input variable adjustment value. In this stage, the relaxation value of the input variable obtained in the first stage is listed as the dependent variable, and the Environmental variable is listed as the independent variable. SFA is used to analyze the influence direction and degree of environmental factors and random disturbance factors on the input relaxation value, and the original input term is adjusted accordingly. The third stage is the operation of adjusted efficiency value, that is, the traditional DEA model is used to evaluate the efficiency using the adjusted input item and the original output item, and then the efficiency analysis, relaxation value analysis and return to scale analysis are implemented. Given the combination of the characteristics of SFA and DEA, the three-stage DEA model has been widely used in the research of transportation industry, finance industry, insurance industry, agriculture, tourism industry, manufacturing industry, scientific and technological innovation, education and other fields, and some scholars continue to improve and deepen research on it.

## 3. Research design

### 3.1. Research Framework

First, we use the three-stage DEA model to analyses Happy Farmhouse. It can better understand the relevant characteristics of its operational efficiency. Second, the Wilcoxon non-parametric test is performed on all kinds of efficiency values in the first and third stages to check whether there are significant differences between the two efficiency values. Finally, the DEA super efficiency model is applied to analyze the efficiency of the input adjustment value and the original output item, and explore the benchmark enterprises in the decision-making unit.

### 3.2. Analysis method

#### 3.2.1. Data envelopment analysis.

CCR-DEA is an efficiency evaluation model developed by Charnes [38] to evaluate the relative efficiency of each decision-making unit. Banker [39]and others removed the restriction that the return to scale should be fixed in the CCR model and proposed the BCC model. When using the DEA model to calculate efficiency, the first thing to consider is the choice of input-oriented and output-oriented DEA models. If the research object belongs to a profitable enterprise and cannot be controlled due to market demand volatility, but input items can be adjusted freely, input orientation should be adopted. Otherwise, output orientation is used. Therefore, the traditional input-oriented DEA model is adopted in this study.

Different from the CCR model, the BCC model assumes that variable returns to scale, that is, the increase of some inputs does not lead to a relative increase of output items. Therefore, the concept of distance function is used to derive a model that can measure pure technical efficiency, efficiency to scale and return to scale. Suppose the unit *j (j = 1,..., n)* uses *i (i = 1,..., m)* input quantity is *x*_*ij*_, whose *r (r = 1,... s)* output is *y*_*rj*_, *h*_*j*_ is the technical efficiency of the *j*th decision unit, *τ*_*r*_ and *ω*_*i*_ are the weights of the *r*th output item and the ith input item respectively, and *μ*_*k*_ is the return to scale index of the kth decision unit, then the linear programming model of the BCC model is expressed as:


$$\text{Max}\;{\text{h}}_{\text{k}}=\sum_{\text{r}=\text{1}}^{\text{s}}\tau_{\text{r}}{\text{y}}_{\text{rk}}-{\text{u}}_{\text{k}}$$

$$\text{s}.\text{t}.\;\sum_{\text{i}=\text{1}}^{\text{m}}\omega_{\text{i}}{\text{x}}_{\text{ik}}=\text{1}$$

$$\sum_{\text{r}=\text{1}}^{\text{s}}\tau_{\text{r}}{\text{y}}_{\text{rk}}-\sum_{\text{i}=\text{1}}^{\text{m}}\omega_{\text{i}}{\text{x}}_{\text{ik}}-{\text{u}}_{\text{k}}\leq \text{0}$$ (1)

$$\tau_{\text{r}}\geq \text{0};\omega_{\text{i}}\geq \text{0};\text{r}=\text{1},\text{2},\cdots \text{s};$$

K=1, 2, ⋯,n; i=1, 2, ⋯,m

For easy calculation and additional information in interpretation, the formula (1) is converted into the dual form as follows:


$$\text{Min}\;{\text{h}}_{\text{k}}=\theta -\epsilon \left(\sum_{\text{i}=\text{1}}^{\text{m}}{\text{s}}_{\text{i}}^{-}+\sum_{\text{r}=\text{1}}^{\text{s}}{\text{s}}_{\text{r}}^{+}\right)$$

$$\text{s}.\text{t}.\;\sum_{\text{j}=\text{1}}^{\text{m}}\lambda_{\text{j}}{\text{x}}_{\text{ij}}-\theta {\text{x}}_{\text{ik}}+{\text{s}}_{\text{i}}^{-}=\text{0}$$

$$\sum_{\text{j}=\text{1}}^{\text{n}}\lambda_{\text{j}}{\text{y}}_{\text{rj}}-{\text{s}}_{\text{r}}^{+}={\text{y}}_{\text{rk}}$$

$$\sum_{\text{j}=\text{1}}^{\text{n}}\lambda_{\text{j}}=\text{1}$$

$$\lambda_{\text{j}},{\text{s}}_{\text{i}}^{-},{\text{s}}_{\text{r}}^{+}\geq \text{0};;\text{r}=\text{1},\text{2},\cdots \text{s};$$

K=1, 2⋯,n;i=1, 2⋯,m  (2)

θ has no positive or negative restrictions, si- and sr + are the relaxation values of the input and output terms, respectively, and ε is a non-Archimedes constant.

If θ = 1, si- = sr+ = 0, it is strong efficiency. If θ = 1, si- ≠ 0 or sr+≠0, it is weak efficiency. If θ < 1, it is not efficiency.

Since there may be more than one relatively efficient DMU, Anderson [40] and others sorted efficient DMU based on the efficiency value of CCR model and proposed the concept of super efficiency. It is measured by removing a certain DMU with an efficiency value of 1 from the efficiency frontier, then constructing a new efficiency frontier based on the remaining DMU, and recalculating the distance from the eliminated DMU to the new efficiency frontier. Since the eliminated DMU is not enveloped by the new efficiency frontier, the calculated new efficiency value will be greater than 1, so that it is easier to sort the efficient DMU, and the efficiency value is the super efficiency value. Its basic mode is expressed as:


$$\theta^{*}=min\theta$$

$$\text{s}.\text{t}.\;\sum_{\text{j}=\text{1},\text{j}\neq \text{k}}^{\text{n}}\lambda_{\text{j}}{\text{x}}_{\text{ij}}+{\text{s}}_{\text{i}}^{-}=\theta {\text{x}}_{\text{ik}},\forall \text{k}\in \text{n}$$

$$\sum_{\text{j}=\text{1},\text{j}\neq \text{k}}^{\text{n}}\lambda_{\text{j}}{\text{y}}_{\text{rj}}-{\text{s}}_{\text{r}}^{+}={\text{y}}_{\text{rk}},\forall \text{k}\in \text{n}$$

$$\sum_{\text{j}=\text{1}}^{\text{n}}\lambda_{\text{j}}=\text{1},\lambda_{\text{j}}\geq \text{0};\;{\text{s}}_{\text{i}}^{-},{\text{s}}_{\text{r}}^{+}\geq \text{0}$$

i=1,2,⋯m;r=1,2,⋯s; j=1,2,⋯n (3)

θ* is the super efficiency value, and s- and s + are the vectors composed of the relaxation values of the input and output terms, respectively.

#### 3.2.2. Random frontier analysis.

In the second stage, environmental factors, statistical interference and management inefficiency are decomposed by the cost boundary function of the stochastic production frontier. Assuming that the ith input relaxation value of the jth decision making unit is sij and subject to k exogenous environmental variables z_j_ = [z_1j_, z_2j_,..., z_kj_], according to the ideas of Battese [41] the relationship between input relaxation value and exogenous environmental variables can be set as follows using stochastic frontier method:


$$s_{\text{ij}}=\;f_{i}\;(z_{j}\;;\;\beta_{i})+{\text{v}}_{\text{ij}}\;+\;{\text{u}}_{\text{ij}}$$ (4)

f_i_ (z_j_; β_i_) is the influence function of ecological variables on input relaxation value, β_i_ is the parameter vector of ecological factors to be estimated, residual term is combination error v_ij_ + u_ij_, v_ij_ ~ N (0, $\sigma_{{\text{v}}_{\text{i}}}^{\text{2}}$) is statistical error, u_ij_ ~ N(u_i_, $\sigma_{{\text{u}}_{\text{i}}}^{\text{2}}$) is management inefficiency, and they are assumed to be independent and uncorrelated. The parameters (β_i_,${\;\sigma }_{{\text{v}}_{\text{i}}}^{\text{2}}$, u_i_, $\sigma_{{\text{u}}_{\text{i}}}^{\text{2}}$ are estimated using the maximum likelihood method.

In order to adjust the variable value of the original input term of the DMU, the conditional estimator of uij is obtained by using formula (5), where Φ and φ are the distribution function and density function of the standard normal distribution, respectively.


$$\text{E}\left({\text{u}}_{\text{ij}}|\epsilon_{\text{i}}\right)=\sigma_{*}\left[\frac{\varphi (\frac{\epsilon_{\text{i}}\lambda }{\sigma })}{\Phi (\frac{\epsilon_{\text{i}}\lambda }{\sigma })}+\frac{\epsilon_{\text{i}}\lambda }{\sigma }\right]$$

$$\epsilon_{\text{i}}={\text{v}}_{\text{ij}}+{\text{u}}_{\text{ij}};\lambda =\frac{\sigma_{\text{u}}}{\sigma_{\text{v}}}$$

$$\sigma^{\text{2}}=\sigma_{u}^{\text{2}}+\sigma_{v}^{\text{2}};\sigma_{*}=\frac{\sigma_{\text{u}}\sigma_{\text{v}}}{\sigma }$$

i=1,2,⋯m;j=1,2,⋯n (5)

Combining formula (4) and formula (5), statistical errors can be separated and conditional estimates of statistical factors can be obtained:


$$\text{E}\left({\text{v}}_{\text{ij}}|{\text{v}}_{\text{ij}}+{\text{u}}_{\text{ij}}\right)=\;{\text{s}}_{\text{ij}}-{\text{z}}_{\text{j}}\beta_{\text{i}}-\text{E}\left({\text{u}}_{\text{ij}}|{\text{v}}_{\text{ij}}+\;{\text{u}}_{\text{ij}}\right)\;;$$ (6)

i=1, 2, …, m; j=1, 2, …, n.

Then the results of SFA are used to adjust the input items, and all DMUs are adjusted to the same environmental conditions. There are two ways to adjust: one is to increase the number of DMU input with good operating environment, so that its operating environment reaches the worst and its luck reaches the worst degree; The other way is to reduce the number of DMUs that deal with bad operating environment to the optimal operating environment and the best luck. This approach follows Fried et al. (2002), who recommend the first adjustment method to avoid potential negative input values that could complicate subsequent analysis [47] The formula is expressed as:


$${\text{x}}_{\text{i}*\text{j}}\;=\;{\text{x}}_{\text{ij}}+\left[{\text{max}}_{\text{j}}\;\left\{{\text{z}}_{\text{j}};\beta_{\text{i}}\right\}-{\text{z}}_{\text{j}}\beta_{\text{i}}\right]+\left[{\text{max}}_{\text{j}}\left\{{\text{v}}_{\text{ij}}\right\}-{\text{v}}_{\text{ij}}\right];\;\text{i}\;=\text{1},\text{2},\ldots ,\text{m};\;\text{j}\;=\;\text{1},\;\text{2},\ldots ,\text{n}.$$ (7)

x_i*j_ is the adjusted value of the actual input x_ij_. The first bracket represents adjusting all DMUs to the same operating environment, and the second bracket represents adjusting all DMUs to the same luck. When both environmental factors and statistical interference items are adjusted to be consistent, only the effect of management inefficiency remains.

### 3.3. Research samples, indicators and data

This study employs a mixed-methods approach, combining both quantitative and qualitative data collection techniques to ensure comprehensive data coverage. Our field research utilized multiple specific instruments, including semi-structured interviews with farmhouse owners and managers to understand operational practices and challenges, on-site verification checklists to document physical infrastructure and service quality, and operational data collection forms for extracting financial and operational metrics. The evaluation indicators of this study were determined based on the homogeneity requirements of DMU proposed by Golany [42], while also referring to relevant indicators in the system. The research focuses on Anji County in Huzhou City, Zhejiang Province, which has cultivated over 4,000 Happy Farmhouses since 2006, including 10 cluster villages/areas. Based on field research and literature analysis, we categorized Happy Farmhouses into three distinct business entity types: individual farmer type (small-scale family-based operations), farmer participation type (hybrid models involving farmer-external investor collaborations), and company cluster type (large-scale operations led by specialized enterprises). Using stratified random sampling from the Zhejiang Provincial Department of Culture and Tourism information system, we selected 50 Happy Farmhouses as research samples (16 individual farmer, 17 farmer participation, and 17 company cluster), ensuring proportional representation of each business type while controlling for geographic distribution across 5 townships and operational duration of at least three years. This sampling strategy enabled comparative analysis across different governance structures, which is central to our research objectives. The evaluation indicators were determined based on homogeneity requirements while incorporating field-verified metrics, with inputs including human resources (high-quality practitioners), material resources (types of tourism elements, number of meals), and financial resources (total investment), while outputs encompass economic benefits (annual operating income, catering income) and social benefits (increase in local employment). All data were obtained from the Zhejiang Provincial Rural Tourism Information System and supplemented by field verification, ensuring both methodological rigor and practical relevance for our efficiency analysis. Descriptive statistics are detailed in Table 1.

**Table 1:** Descriptive statistics of relevant variables.

item	unit	sample capacity	Min	Max	average	Standard deviation
high-quality practitioners	person	50	0	28	8. 926684	7. 0736618
types of tourism elements	unit	50	1	8	3. 42	1. 4844528
number of meals	unit	50	30	250	71. 96	47. 696524
total investment	Ten thousand yuan	50	10	8000	1336. 94	1996. 6412
Annual operating income	Ten thousand yuan	50	30	20000	3589. 54	4236. 9298
catering income	Ten thousand yuan	50	30	1200	398. 7	232. 42515
increase the number of local employees	person	50	4	220	81. 6	65. 408562

## 4. Result analysis

### 4.1. Static analysis of farm operations efficiency

#### 4.1.1. Stage I: DEA efficiency under original input-output data.

In this study, the input-oriented BCC model and MaxDEA8 software were used to measure the relative operational efficiency of 50 happy farmhouses in Huzhou area of Zhejiang Province in 2023. Among them, only 14 happy farmhouses had effective DEA, and 36 had decision units that produced input relaxation value. (As shown in Table 2).

**Table 2:** Efficiency analysis table of the first stage.

DMU	CRS	VRS	Scale	RTS	Efficiency
DMU001	0. 6940721	0. 993843	0. 698372	Decreasing	Not Efficiency
DMU002	0. 4117572	0. 682918	0. 602938	Increasing	Not Efficiency
DMU003	0. 902832	1	0. 902832	Increasing	Not Efficiency
DMU004	0. 5268891	0. 781933	0. 673829	Decreasing	Not Efficiency
DMU005	0. 9233564	0. 984922	0. 937492	Decreasing	Not Efficiency
DMU006	0. 7733419	0. 874839	0. 883982	Decreasing	Not Efficiency
DMU007	0. 948732	1	0. 948732	Decreasing	Not Efficiency
DMU008	0. 8594047	0. 983493	0. 873829	Increasing	Not Efficiency
DMU009	0. 8947841	0. 984932	0. 908473	Increasing	Not Efficiency
DMU010	0. 4352484	0. 489383	0. 889382	Increasing	Not Efficiency
DMU011	0. 4539188	0. 673827	0. 673643	Decreasing	Not Efficiency
DMU012	0. 998372	1	0. 998372	Decreasing	Not Efficiency
DMU013	0. 5453155	0. 673822	0. 809287	Increasing	Not Efficiency
DMU014	0. 8088089	0. 908374	0. 890392	Increasing	Not Efficiency
DMU015	0. 3831442	0. 564783	0. 678392	Increasing	Not Efficiency
DMU016	0. 2714213	0. 473822	0. 572834	Decreasing	Not Efficiency
DMU017	0. 8655311	0. 940392	0. 920394	Decreasing	Not Efficiency
DMU018	1	1	1	Constant	Weak Efficiency
DMU019	0. 5313272	0. 704938	0. 753722	Increasing	Not Efficiency
DMU020	0. 903945	1	0. 903945	Decreasing	Not Efficiency
DMU021	1	1	1	Constant	Strong Efficiency
DMU022	1	1	1	Constant	Strong Efficiency
DMU023	0. 809182	1	0. 809182	Decreasing	Not Efficiency
DMU024	0. 7169907	0. 789382	0. 908293	Increasing	Not Efficiency
DMU025	1	1	1	Constant	Strong Efficiency
DMU026	1	1	1	Constant	Strong Efficiency
DMU027	0. 9226544	0. 993821	0. 928391	Decreasing	Not Efficiency
DMU028	1	1	1	Constant	Strong Efficiency
DMU029	0. 2315865	0. 892833	0. 259384	Increasing	Not Efficiency
DMU030	0. 6402614	0. 703984	0. 909483	Increasing	Not Efficiency
DMU031	1	1	1	Constant	Weak Efficiency
DMU032	0. 8092833	1	0. 809283	Decreasing	Not Efficiency
DMU033	1	1	1	Constant	Strong Efficiency
DMU034	1	1	1	Constant	Strong Efficiency
DMU035	0. 7536471	1	0. 753647	Increasing	Not Efficiency
DMU036	0. 7715255	0. 889384	0. 867483	Increasing	Not Efficiency
DMU037	0. 8660251	0. 928341	0. 932874	Decreasing	Not Efficiency
DMU038	0. 7363819	0. 789403	0. 932834	Decreasing	Not Efficiency
DMU039	1	1	1	Constant	Strong Efficiency
DMU040	1	1	1	Constant	Strong Efficiency
DMU041	0. 7352024	0. 809384	0. 908347	Increasing	Not Efficiency
DMU042	0. 7730140	0. 829384	0. 932034	Decreasing	Not Efficiency
DMU043	0. 6846166	0. 789309	0. 867362	Decreasing	Not Efficiency
DMU044	0. 2872397	0. 809872	0. 354673	Increasing	Not Efficiency
DMU045	0. 7197617	0. 809283	0. 889382	Increasing	Not Efficiency
DMU046	0. 9200053	0. 935465	0. 983473	Increasing	Not Efficiency
DMU047	1	1	1	Constant	Strong Efficiency
DMU048	1	1	1	Constant	Strong Efficiency
DMU049	1	1	1	Constant	Strong Efficiency
DMU050	0. 5842344	0. 865748	0. 674832	Increasing	Not Efficiency
Avg.	0. 7818762	0. 8910362	0. 864835		

#### 4.1.2. Stage II: Environmental analysis and adjustment affecting input-output efficiency.

Frontier 4.1 software is used to analyses the SFA of the environmental factors affecting the operational efficiency of the Happy Farmhouse. That is, perform regression analysis with three environmental variables with logarithm as the independent variable and the relaxation of four inputs in the DMU measured in stage I is taken as the dependent variable with logarithm. The results are shown in Table 3.

**Table 3:** The second stage random frontier regression estimation results.

	high-quality practitioners		types of tourism elements		number of meals		total investment	
	β	t	β	t	β	t	β	t
Constant	−124. 773	−122. 566	−0. 003	−0. 015	−1. 028	−0. 087	−0. 017	−1. 675
land area	0. 304	5. 691	0. 000	0. 007	0. 003	0. 063	0. 000	2. 096
The level of economic development	−0. 1080	−2. 547	−0. 000	−0. 019	−0. 001	−0. 03713	−0. 000	−0. 821
policy innovation	−0. 1809	−4. 660	−0. 000	-. 0036	-. 0007	−0. 113	−0. 000	−0. 975
*σ* ^*2*^	20383. 0	20400. 0	12. 914	13. 442	472. 362	2020. 0	10. 974	2. 454
γ	0. 999	218000. 0	0. 999	3280000.	0. 9999	546000. 0	0. 999	386000000.
Log likelihood function	−284. 574	—	−101. 332	—	−169. 575	—	−59. 9973	—
LR test of the one-sided error	22. 4518	—	20. 0640	—	63. 8889	—	34. 7714	—

According to the SFA regression results in Table 3, all three environmental variables demonstrate statistically significant effects on input slacks at the 5% level, confirming the validity of our variable selection. Specifically, the analysis reveals distinct directional impacts: land area exhibits a significant positive coefficient on the slack of high-quality practitioners (β = 0.304, t = 5.691), indicating that larger operational areas correlate with increased input redundancy. In contrast, both the level of economic development (β = −0.1080, t = −2.547) and policy innovation (β = −0.1809, t = −4.660) show significant negative coefficients on the same input slack, suggesting these factors contribute to reducing resource waste and improving input utilization efficiency. The highly significant γ values (approaching 1.0) and the conclusive LR one-sided error tests (all exceeding critical values) further validate that management inefficiency, rather than random noise, dominates the performance variations, thus confirming the necessity of our second-stage SFA analysis.

A positive regression coefficient indicates that increasing the environmental variable value will increase the input slack value (input redundancy), resulting in waste or insufficient output of each input variable. Conversely, a negative regression coefficient indicates that increasing the value of environmental variables is conducive to reducing the amount of input relaxation, that is, to reducing the waste of each input variable or increasing the output.

(1) The level of economic development. The regression coefficients of the economic development level of the four input relaxation variables are all negative, indicating that the economic development level measured by regional per capita GDP reduces the relaxation value of all input variables and improves the efficiency of fund use, which is consistent with the findings of Chen et al. [43] and Migue et al. [44] With the increase of per capita GDP, on the one hand, there will be greater consumption power for infrastructure services, such as transportation, public facilities, schools, and medical and health facilities, and the demand will increase with the increase of income. On the other hand, it can also provide sufficient financial support for infrastructure investment and construction, promote the supply of infrastructure, and drive the improvement of the input-output efficiency of infrastructure.
(2) Land area. The land area regression coefficients of the four input relaxation variables are all positive, indicating that the increase of land area has a reverse effect on the efficiency of these four investments, reflecting that the larger the farmland management area, the greater the investment input should be.
(3) Policy innovation. The policy innovation regression coefficients of the four input relaxation variables are all negative, indicating that the degree of regional policy innovation support reduces the relaxation value of input variables and improves the technical efficiency of the management performance of farmhouse. This is consistent with the findings of Hu et al. [45] and Wang and Hao [46]. The introduction of policy innovation can bring advanced management ideas and promote the application of new technologies [47], which is conducive to achieving higher output efficiency with less input cost.

The above results show that the three environmental variables have different directions of influence on the efficiency of happy farmhouse, which may lead to overestimation of the efficiency value of DMUs in better environment or luck, and underestimation of the efficiency value of DMUs in worse environment or luck. Therefore, the environmental impact value of relaxation value of each input term can be obtained by means of SFA regression equation through the analysis of the second stage. The mixed error term is obtained from formula (4), and then the random interference term is obtained by separating the management inefficiency value from formula (5) and formula (6), and then the environmental adjustment value and random adjustment value are further obtained. Finally, the adjustment value of the input term is obtained from formula (7), so that all DMUs are in the same environment.

#### 4.1.3. Stage III: Empirical results of DEA after input adjustment.

According to the SFA empirical results of stage II, the MaxDEA8 software is used to bring the adjusted input value and the original output into the model again for efficiency measurement, and it was divided into three categories for statistical analysis, and the results were shown in Table 4.

**Table 4:** Efficiency analysis table of the third stage.

DMU	CRS	VRS	Scale	RTS	Efficiency	Ares
DMU001	0. 79632006	1	0. 79632006	Decreasing	Not Efficiency	individual farmer
DMU002	0. 49653314	0. 7426636	0. 66858410	Increasing	Not Efficiency	
DMU003	1	1	1	Increasing	Weak Efficiency	
DMU004	0. 67692307	0. 8788135	0. 77026926	Decreasing	Not Efficiency	
DMU005	1	1	1	Decreasing	Weak Efficiency	
DMU006	0. 875	0. 9473684	0. 92361111	Decreasing	Not Efficiency	
DMU007	1	1	1	Decreasing	Weak Efficiency	
DMU008	0. 9	1	0. 9	Increasing	Not Efficiency	
DMU009	1	1	1	Increasing	Weak Efficiency	
DMU010	0. 48875	0. 53836828	0. 90783579	Increasing	Not Efficiency	
DMU011	0. 54428881	0. 77112299	0. 70583917	Increasing	Not Efficiency	
DMU012	1	1	1	Constant	Strong Efficiency	
DMU013	0. 66160886	0. 74724241	0. 88540057	Increasing	Not Efficiency	
DMU014	1	1	1	Increasing	Weak Efficiency	
DMU015	0. 48260162	0. 65243339	0. 73969485	Increasing	Not Efficiency	
DMU016	0. 45100426	0. 68735510	0. 65614448	Increasing	Not Efficiency	
DMU017	1	1	1	Constant	Weak Efficiency	farmer participation
DMU018	1	1	1	Constant	Weak Efficiency	
DMU019	0. 58035714	0. 75707636	0. 76657675	Increasing	Not Efficiency	
DMU020	0. 86671734	1	0. 86671734	Decreasing	Not Efficiency	
DMU021	1	1	1	Constant	Strong Efficiency	
DMU022	1	1	1	Constant	Strong Efficiency	
DMU023	1	1	1	Decreasing	Strong Efficiency	
DMU024	0. 78670026	0. 84205384	0. 93426362	Increasing	Not Efficiency	
DMU025	1	1	1	Constant	Strong Efficiency	
DMU026	1	1	1	Constant	Strong Efficiency	
DMU027	1	1	1	Constant	Strong Efficiency	
DMU028	1	1	1	Constant	Strong Efficiency	
DMU029	0. 27777777	1	0. 27777777	Increasing	Not Efficiency	
DMU030	1	1	1	Increasing	Strong Efficiency	
DMU031	1	1	1	Constant	Weak Efficiency	
DMU032	1	1	1	Decreasing	Weak Efficiency	
DMU033	1	1	1	Constant	Strong Efficiency	
DMU034	1	1	1	Constant	Strong Efficiency	company cluster
DMU035	1	1	1	Increasing	Strong Efficiency	
DMU036	0. 80409356	0. 90486127	0. 88863739	Increasing	Not Efficiency	
DMU037	1	1	1	Constant	Strong Efficiency	
DMU038	1	1	1	Constant	Strong Efficiency	
DMU039	1	1	1	Constant	Strong Efficiency	
DMU040	1	1	1	Constant	Strong Efficiency	
DMU041	1	1	1	Increasing	Weak Efficiency	
DMU042	1	1	1	Constant	Weak Efficiency	
DMU043	0. 70104907	0. 80148077	0. 87469231	Increasing	Not Efficiency	
DMU044	0. 42574257	1	0. 42574257	Increasing	Not Efficiency	
DMU045	0. 78804347	0. 8662737	0. 90969341	Increasing	Not Efficiency	
DMU046	0. 93417532	0. 95179613	0. 98148678	Increasing	Not Efficiency	
DMU047	1	1	1	Constant	Strong Efficiency	
DMU048	1	1	1	Constant	Strong Efficiency	
DMU049	1	1	1	Constant	Strong Efficiency	
DMU050	1	1	1	Increasing	Strong Efficiency	
Avg	0. 83356720	0. 9294482	0. 8915285

(1) From Table 4, there is a significant difference in the operational efficiency of the 50 happy farmhouses before and after the input adjustment. In terms of absolute numbers, the number of DMUs with constant returns to scale in phases I and III is 14 and 20, respectively, for a total increase of 6 units. The DMUs with increasing returns to scale were 19 and 22, for a total increase of three; The number of DMUs with decreasing returns to scale was 17 and 8 each, for a total reduction of 9; Among the 19 Not Efficiency DMUs, 5 of them are due to scale inefficiencies, while the remaining 14 Not Efficiency ones are due to both pure technical efficiency and scale inefficiencies. The average value of the overall technical efficiency of the decision-making units increased from 0. 7818–0. 8335, a total increase of 6. 61 per cent. The average value of pure technical efficiency increased from 0. 8910–0. 9294, for a total improvement of 4. 3109 per cent. The average value of scale efficiency increased from 0. 8648–0. 8915, for a total improvement of 3. 0864 per cent. After removing the environmental impact, the pure technical efficiency of the provinces, autonomous regions and municipalities has increased to some extent, while the scale efficiency has decreased significantly. The adjusted pure technical efficiency is significantly higher than the scale efficiency, indicating that the lower comprehensive technical efficiency is mainly caused by the scale inefficiency.
(2) From the perspective of the three different types of business entities, DMU1-DMU16, belong to the individual farmer type of business entities, and their overall efficiency is low, with a total of 10 Not Efficiency, 5 DEA weakly efficient, and only one DEA strong Efficiency. DMU17-DMU33 belong to the farmer participation type of business entities, while their overall efficiency values are relatively high, with the existence of 9 DEA strong Efficiency, 4 DEA weak Efficiency and 4 Not Efficiency happy farmhouses. DMU34-DMU50 belong to the company cluster type of business entities and have the highest overall efficiency values, with the presence of 10 DEA strong Efficiency, 2 DEA weak Efficiency, and 5 Not Efficiency. This suggests that through the company cluster model of the happy farmhouse, it has a stronger supply chain supply capacity, higher efficiency of business operation, and the clustered area can provide a better business environment and customer flow environment for the local happy farmhouses compared to non-clustered areas.
(3) In this study, the MaxDEA8 software was used to measure the super-efficiency using the CCR model. The analysis of the DEA super-efficiency model yielded specific scores for the 50 happy farmhouses (See Table 5), focusing on the 31 DMUs that were in a relatively efficient position, i.e., those whose original technical efficiency value was equal to 1. Six DMUs were found to have an efficiency value greater than 2. They are 039 (24. 9382912), 034 (16. 8759667), 022 (3. 987862), 040 (2. 983749), 021 (2. 764891), and 048 (2. 093847).

**Table 5:** Results of performance evaluations.

DMU	effective	ranking	DMU	effective	ranking
DMU001	0. 79632006	36	DMU026	1. 673891	13
DMU002	0. 496533142	45	DMU027	1. 0938475	25
DMU003	1. 002736	30	DMU028	1. 756382	11
DMU004	0. 676923077	40	DMU029	0. 277777778	50
DMU005	1. 27319	23	DMU030	1. 37181	18
DMU006	0. 875	33	DMU031	1. 7484595	12
DMU007	1. 28391	21	DMU032	1. 2983	19
DMU008	0. 9	32	DMU033	1. 456382	15
DMU009	1. 00293	29	DMU034	16. 8759667	2
DMU010	0. 48875	46	DMU035	1. 93812	9
DMU011	0. 544288815	44	DMU036	0. 804093567	35
DMU012	1. 008359	28	DMU037	1. 289109	20
DMU013	0. 661608863	41	DMU038	1. 00987632	27
DMU014	1. 39821	16	DMU039	24. 9382912	1
DMU015	0. 482601626	47	DMU040	2. 983749	4
DMU016	0. 45100426	48	DMU041	1. 118293	24
DMU017	1. 392034	17	DMU042	1. 28374981	22
DMU018	1. 8298347	10	DMU043	0. 701049078	39
DMU019	0. 580357143	43	DMU044	0. 425742574	49
DMU020	0. 866717348	34	DMU045	0. 788043478	37
DMU021	2. 764891	5	DMU046	0. 934175323	31
DMU022	3. 987862	3	DMU047	1. 9803847	8
DMU023	1. 02983	26	DMU048	2. 093847	6
DMU024	0. 786700269	38	DMU049	1. 987362	7
DMU025	1. 4782392	14	DMU050	0. 638410436	42

**Fig 1:**
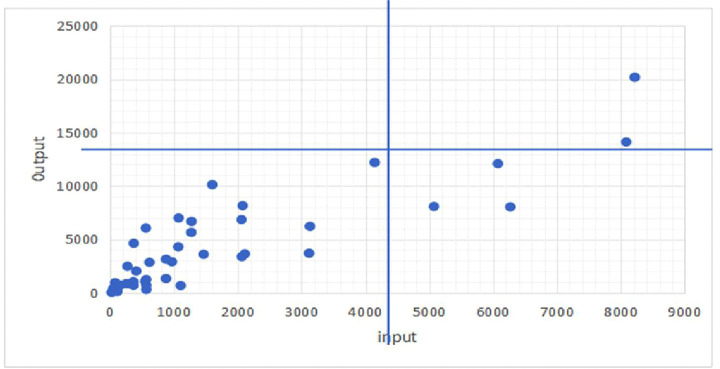
Quadrant Analysis.

(4) In order to further find the direction of improving the operational efficiency of the happy farmhouse, this article uses PCA to reduce the dimensionality of input and output data from various decision-making units, and then through the way of quadrant diagrams presenting the spatial distribution of decision-making units, which can be divided into the four quadrants of ABCD. Quadrant A: high inputs, high outputs; Quadrant B: low inputs, high outputs; Quadrant C: low inputs, low outputs; Quadrant D: high inputs, low outputs. As shown in Fig 1. Most of the farmhouses in the sample cases are in the C phenomenon, i. e., low-input, low-output mode, which is also due to the business nature of farmhouses in this province. The farmhouse itself is a small business, the threshold of entry is relatively low, and the small yard in the farmer’s house can be opened for business after a little bit of organizing and cleaning.

### 4.2. Dynamic analysis of the efficiency of rural tourism management

To further portray the longitudinal changes of DMU efficiency over time and explore the reasons for the changes in the efficiency of happy farmhouses, this paper selects the panel data of 50 happy farmhouses in the Huzhou area of Zhejiang Province and utilizes the MaxDEA8 software to solve the super-efficiency index to get the 50 happy farmhouses’ productivity (TFP) and its changes.

#### 4.2.1. Trends in the M-Productivity Index and its decomposition.

Table 6 reports the changes in the productivity index, technical progress and combined technical efficiency of happy farmhouses in the Huzhou region over the last 10 years. From 2014 to 2023, the overall average annual growth rate of TFP in the region was 3. 1%, but the fluctuation was significant, with the highest being 21. 5%, which occurred from 2022 to 2023; The lowest is −3. 7%, which occurred from 2019 to 2020. From 2014 to 2016, TFP showed a slow upward trend in stages, with an average annual growth rate of 2. 8%. From 2016 to 2019, TFP showed a clear upward trend, with an average annual growth rate of 8. 6%; From 2019 to 2021, TFP showed a downward trend, with an average annual growth rate of −3. 0%. From 2021 to 2023, TFP showed a clear upward trend. This result indicates that since 2014, under the extensive development model that emphasizes “scale expansion”, the investment in rural infrastructure construction in the Huzhou area has grown rapidly, and the number and location of rural leisure activities have quickly increased year-on-year. However, the M productivity index is less than 1, indicating that this growth is inefficient and lacks quality assurance; Since the proposal of rural revitalization in 2017, various policy measures favorable to the development of rural tourism have been introduced in Zhejiang Province, resulting in a significant increase in the number of rural tourism tourists. This has prompted investment in rural tourism in Zhejiang Province to shift towards a cluster development model, leading to rapid growth in operational efficiency. From 2016 to 2019, the M productivity index was significantly greater than 1, indicating that the growth of TFP during this period is guaranteed high quality. At the end of 2019, the epidemic’s impact significantly affected China’s entire tourism economy. There was no unexpected negative growth in the operation of rural tourism. However, as the epidemic subsided, the rise of short-distance rural tourism led to a rapid upward cycle in the operating performance of rural tourism.

**Table 6:** Efficiency and Productivity Index of Happy Farmhouse in Huzhou.

Year	TFP	TC	TEC	PTEC	SEC
2014-2015	0. 963	0. 971	0. 992	1. 010	0. 982
2015-2016	0. 976	0. 979	0. 997	1. 006	0. 991
2016-2017	1. 092	1. 084	1. 008	1. 005	1. 003
2017-2018	1. 148	1. 094	1. 05	1. 04	1. 009
2018-2019	1. 215	1. 167	1. 041	1. 046	0. 995
2019-2020	0. 984	0. 995	0. 989	1. 002	0. 987
2020-2021	0. 969	0. 982	0. 987	0. 992	0. 995
2021-2022	0. 971	0. 987	0. 984	0. 990	0. 994
2022-2023	0. 993	0. 998	0. 995	1. 006	0. 989
average	1. 031	1. 026	1. 004	1. 011	0. 994

Note: TFP = TC*TEC = TC*PTEC*SEC

Further examination of the reasons for the change in TFP shows that the TFP improvement mainly stems from technological progress. Over the past 10 years, technological progress’s average annual growth rate has been 2.6%, contributing 83.9% to TFP and exhibiting the same trend as the M productivity index. With the implementation of the rural revitalization policy in 2017, investment and construction in rural infrastructure have been vigorously promoted, and the average annual growth rate of technological progress has significantly increased; After 2019, in addition to external factors such as the COVID-19, Zhejiang Province has basically achieved the transformation of new rural areas, completed the updating and replacement of basic hardware equipment in rural areas, gradually released institutional dividends. The speed of technological progress will decrease. Its contribution to TFP will also decrease accordingly.

#### 4.2.2. TFP analysis of different business models.

Further dividing the management model of rural tourism into individual farmers, farmer participation and company clusters, the TFP changes of 50 rural tourism enterprises in the Huzhou area from 2014 to 2023 were calculated and compared, as shown in Table 7. Overall, the company cluster type has the highest mean value of TFP (1,058), and the individual farmer type has a lower TFP (0,998). The evolution of the above pattern differences can be roughly divided into two stages: In 2014–2016, the TFP of all three regions declined, and the difference was not noticeable; in 2016–2019, the TFP of each area was amid fluctuation and increase, and the regional difference was gradually expanding. These changes may be attributed to the large-scale and rapid investments made by local governments with the help of land finance funds, leading to the widespread duplication of infrastructure construction as well as the problems of focusing on inputs rather than management and care, and development rather than protection; after 2017, the construction of urbanization around the world has been transformed into high-quality development, and the gap between the efficiency of infrastructure investment in various regions has been enlarged alongside the general increase in efficiency.

**Table 7:** TFP analysis of different business models.

type	index	Avg
individual farmer	TFP	0. 998
	TC	1. 006
	TEC	0. 992
	PTEC	1. 025
	SEC	0. 968
farmer participation	TFP	1. 027
	TC	1. 028
	TEC	0. 999
	PTEC	1. 002
	SEC	0. 997
company cluster	TFP	1. 058
	TC	1.053
	TEC	1. 004
	PTEC	1. 003
	SEC	1. 001

Further decomposition of TFP shows that the average annual growth rates of technological progress for individual farmers, farmer participation, and company clusters are 0. 6%, 2. 8%, and 5. 3%, respectively, are higher than the average annual growth rates of their combined technological efficiencies of −0. 8%, −0. 1%, and 4%, respectively, suggest that the impetus for the growth of regional TFP comes mainly from technological progress. Among them, technological progress in company cluster types has the most obvious role in promoting TFP, which is because their higher level of economic development gives them a greater advantage in management innovation, technological progress, and the introduction of advanced equipment and talents in urban infrastructure investment; while the continuous deterioration of the scale efficiency of the individual farmer type has led to the largest annual decline in the comprehensive technical efficiency, which may be attributed to the weak financial resources of the government of the individual farmer type areas, coupled with the low level of marketization, the public finance budget is mainly used to maintain recurrent expenditure (basically “food finance”). The participation of social capital is low. This may be because the governments of individual farmer-type areas have weak financial resources. The public finance budget is mainly used to maintain recurrent expenditures (basically belonging to the “food finance”), coupled with the low level of marketization and the low degree of participation of social capital, and the funds for the construction of urban infrastructure have been seriously insufficient for many years, so that the improvement of the efficiency of the scale of investment has been weak.

## 5. Conclusion and Suggestions

### 5.1. Conclusion

This article uses a three-stage DEA model to empirically analyse the efficiency of happy farmhouses in the Huzhou area. The research conclusions are as follows: One is based on efficiency value analysis, where individual farmhouses have both efficiency and non-efficiency trends. The analysis reveals that the Happy Farmhouse sector overall exhibits technical inefficiency. At the individual business level, this inefficiency stems primarily from two sources: most operations suffer from deficiencies in both managerial practices (pure technical inefficiency) and operational scale (scale inefficiency), while a smaller proportion struggle solely with suboptimal scale. This pattern of combined technical and scale challenges aligns with the broader industry context. The overall non-DEA effectiveness is caused by the combination of pure technical efficiency and scale efficiency being non-DEA efficiency, which is consistent with the industry situation. First, based on efficiency analysis, the operational efficiency of Happy Farmhouses exhibit both efficiency and non-efficiency. From the operational models, the company cluster model includes 24 DEA-efficiency entities, representing the highest efficiency level, while the individual farmer model has only 7 DEA-efficiency entities, indicating the lowest efficiency level. This disparity is validated across a decade of panel data from 2014 to 2023. Second, the slack value analysis reveals that DEA-efficiency farmhouse operations show zero slack values in all variables, whereas non-DEA-efficiency ones exhibit non-zero slack values in at least one variable, indicating prevalent issues of input redundancy and output deficiency in their operations.

Third, based on super efficiency analysis, the research sample was sorted by efficiency value, and it was found that the efficiency value of the 039th DMU was the highest. This not only helps the government regulatory department to evaluate the sample based on efficiency, but also identifies benchmarks, becoming a model for other inefficient decision-making units to learn from. The fourth is based on SFA regression analysis. Positive regression coefficients indicate that environmental variables are unfavorable for input variables, leading to an increase in input slack and a decrease in efficiency. Negative regression coefficients indicate that environmental variables are favorable for input variables, resulting in a decrease in input slack and an improvement in efficiency. In the regression analysis of economic development level, the coefficients of the four input slack variables are all negative, indicating that the economic development level measured by regional per capita GDP reduces the slack values of various input variables and improves the efficiency of fund utilization. In the regression analysis of land occupation area, the coefficients of the four input relaxation variables are all positive, indicating that an increase in land occupation area has a reverse effect on the efficiency of these four investments, reflecting that the larger the area of rural tourism management, the greater the increase in investment in all aspects; In the innovation policy regression analysis, the regression coefficients of the four input slack variables are all negative, indicating that the degree of regional policy innovation support reduces the slack value of input variables and improves the technical efficiency of rural tourism management performance. The fifth is based on the observation of the Wilcoxon test results of the efficiency values in the first and third stages. There is a significant difference in the efficiency values between the two stages. After excluding the influence of random interference and environmental variables, the non-DEA efficiency source is management inefficiency, which highlights the individual’s internal management level and ability in rural tourism; At the same time, combined with the observation of the average efficiency values of various types of rural tourism as a whole, compared to the first stage, the efficiency values of the third stage have all increased in absolute quantities. This also indicates that, compared to traditional models, the analysis results of the three-stage DEA model can more accurately reflect the real situation.

### 5.2. Suggestions

Firstly, considering that the overall level of the industry is inefficient, which is caused by both pure technical and scale efficiency being unproductive, it is necessary first to clarify the relationship and strengthen centralized management in industry governance. Due to historical reasons, many local industries have placed happy farmhouse tourism under the jurisdiction of the agricultural sector rather than the tourism sector, resulting in a situation where development assistance comes from the farming sector and business statistics belong to the tourism sector. This leads to a lack of professional and targeted guidance for industry development and scale control. Given the service market and target audience of happy farmhouse tourism, combined with guiding the orderly and sustainable development and planning of the industry, it is reasonable to place it under the jurisdiction of the tourism department. On one hand, scale control and adjustment are carried out through establishment approval and market competition among internal formats of rural tourism. On the other hand, by leveraging the experience, technology, and systems accumulated by the tourism department, the pure technical efficiency of the industry can be improved. For example, developing a scientific rating system for rural tourism can promote the improvement of the service level of the entire sector, increase the promotion and application of standard technologies for enterprises, and promote the overall efficiency of the industry by relying on the practical improvement of rural tourism technology efficiency.

The second is to use policy guidance to promote the intensive development of the happy farmhouse. In response to the characteristics of small size, scattered distribution, and susceptibility to environmental impact of happy farmhouse, we will promote relevant social support with a policy orientation, create a good business environment from the outside, take the path of intensive development, improve the overall risk resistance ability of the industry, reduce operating costs, expand market awareness, and spread the effectiveness of promotional activities, thereby improving the overall efficiency of the industry. The prominent manifestation of intensive development is the formation of industrial clusters of a particular scale within a specific regional scope, generating agglomeration effects. Huzhou farmhouse tourism has already formed a farmhouse tourism cluster, represented by Moganshan and Changxing in Huzhou, which is a beneficial exploration of the path of intensive development.

Thirdly, considering that the main reason for the non-DEA effectiveness of most individuals in terms of technical efficiency is focused on pure technical efficiency, which refers to the internal management factors of happy farmhouse individuals, it is necessary to strengthen and solidify the talent strategy in improving individual efficiency, that is, to build a high-quality team of happy farmhouse’s managers. The urgent task is to effectively enhance the management ability and professional competence of happy farmhouse’s managers. Currently, using industry associations as a platform to promote industry exchange and learning is one of the effective ways to acquire professional knowledge and skills in business management and enrich personal cultural literacy. In the face of increasingly fierce market competition, the happy farmhouse must prioritize survival, not only clarifying business goals and motivations, but also valuing business indicators and guiding business behavior with business data as the guide. Related management capabilities have a positive impact on business performance, which helps to estimate input items reasonably in the early stage, reduce excess investment, avoid blind investment, increase output quantity in operation, and avoid weak output. For example, the influence of professional knowledge application ability will play a role in the early stage of construction and daily operations. Combining SFA analysis results, reasonably determining the operating land area can reduce the investment in renovation and decoration costs, as well as the investment in hiring full-time employees; The ability to use online platforms is currently one of the indispensable business technologies, which can expand reservation channels through third-party platforms, improve reputation through social media, and create unique brands to attract a broader range of customers. This greatly benefits the output items such as the number of overnight guests, operating income, catering income, and other supporting income. To a certain extent, the cultural literacy and aesthetic ability of happy farmhouse’s managers determine the taste and charm of rural tourism products, which helps to build product characteristics, form unique segmented markets, and thus improve business performance.

### 5.3. Research limitations and future directions

This study applies a three-stage DEA-Tobit model to analyze the operational efficiency of happy farmhouse tourism. However, it has limitations in terms of sample coverage. While Zhejiang Huzhou was selected as a representative case of happy farmhouse tourism in China, data from a single region may not fully reflect the regional heterogeneity of operational efficiency across China’s diverse rural areas with varying resource endowments and development models. Consequently, the generalizability of the conclusions requires further validation.

Future research could expand in two directions: First, broadening the scope of the study. Conduct cross-regional comparative analyses by selecting happy farmhouse tourism destinations in eastern, central, and western China to capture regional differences. This would help build a multi-dimensional efficiency evaluation framework that accounts for geographical and socio-economic disparities. Second, deepening research dimensions. Beyond efficiency measurement, explore how emerging factors—such as digital technology adoption, cultural-tourism integration, and policy innovation—impact operational efficiency. Such insights could provide forward-looking strategies for enhancing rural tourism quality and synergizing with rural revitalization goals. These advancements would offer more comprehensive and actionable policy recommendations for sustainable rural development.

### 5.4. Future research could expand in two primary directions

First, the scope of study will be broadened through cross-regional comparative analyses. Selecting happy farmhouse tourism destinations across eastern, central, and western China would help capture regional disparities. This approach would facilitate the construction of a multidimensional efficiency evaluation framework that accounts for geographical and socio-economic variations.

Second, deepen research dimensions by exploring mechanisms through which emerging factors, such as digital technology adoption, cultural-tourism integration, and policy innovation, impact operational efficiency. Such insights could yield forward-looking strategies to enhance rural tourism quality while aligning with rural revitalization goals. These advancements would provide comprehensive and actionable policy recommendations for sustainable rural development.
